# On the need for bundle-specific microstructure kernels in diffusion MRI

**DOI:** 10.1016/j.neuroimage.2019.116460

**Published:** 2020-03

**Authors:** Daan Christiaens, Jelle Veraart, Lucilio Cordero-Grande, Anthony N. Price, Jana Hutter, Joseph V. Hajnal, J-Donald Tournier

**Affiliations:** a Centre for the Developing Brain, School of Biomedical Engineering & Imaging Sciences, King’s College London, London, UK; b Department of Electrical Engineering, ESAT/PSI, KU Leuven, Leuven, Belgium; c Centre for Biomedical Imaging, NYU School of Medicine, New York, NY, USA; d iMinds – Vision Lab, University of Antwerp, Antwerp, Belgium

**Keywords:** Diffusion MRI, Microstructure imaging, Model validation, Multi-fascicle models, Fibre orientation distribution

## Abstract

Probing microstructure with diffusion magnetic resonance imaging (dMRI) on a scale orders of magnitude below the imaging resolution relies on biophysical modelling of the signal response in the tissue. The vast majority of these biophysical models of diffusion in white matter assume that the measured dMRI signal is the sum of the signals emanating from each of the constituent compartments, each of which exhibits a distinct behaviour in the *b*-value and/or orientation domain. Many of these models further assume that the dMRI behaviour of the oriented compartments (e.g. the intra-axonal space) is identical between distinct fibre populations, at least at the level of a single voxel. This implicitly assumes that any potential biological differences between fibre populations are negligible, at least as far as is measurable using dMRI. Here, we validate this assumption by means of a voxel-wise, model-free signal decomposition that, under the assumption above and in the absence of noise, is shown to be rank-1. We evaluate the effect size of signal components beyond this rank-1 representation and use permutation testing to assess their significance. We conclude that in the healthy adult brain, the dMRI signal is adequately represented by a rank-1 model, implying that biologically more realistic, but mathematically more complex fascicle-specific microstructure models do not capture statistically significant or anatomically meaningful structure, even in extended high-*b* diffusion MRI scans.

## 1 Introduction

Probing tissue microstructure with *in vivo* magnetic resonance imaging (MRI) on a scale orders of magnitude below the imaging resolution relies on biophysical modelling of the signal response in intra- and extra-cellular space. In diffusion-weighted MRI (dMRI), the signal is sensitized to the thermal motion of water molecules on length scales that commensurate with various cell dimensions (Le Bihan et al., 1986; Callaghan et al., 1988). Hence, dMRI provides a non-invasive, yet indirect, probe into the tissue micro-environment, despite the coarse spatial resolution of MRI (Alexander et al., 2019; Novikov et al., 2019). Analysis of dMRI data on the macroscopic (voxel) level has shown sensitivity to several microstructural changes, associated with development, ageing, and disorder of the human brain, and of white matter in particular (Neil et al., 2002; Horsfield and Jones, 2002; Sundgren et al., 2004; Sullivan and Pfefferbaum, 2006). However, a more specific interpretation of the signal in terms of brain microstructure relies on the development and validation of a suitable biophysical model (Jelescu and Budde, 2017).

The vast majority of these biophysical white matter models derive from a model of multiple non-exchanging compartments that contribute linearly to the measured dMRI signal. Each compartment is assumed to exhibit a distinct behaviour in the *b*-value and/or orientation domain, characterised by a *kernel* or *response function* that encapsulates the expected signal behaviour that would be measured for that compartment in isolation. Many models further impose the constraint that the various compartments in a single voxel share the same orientation structure; for example, the extra-cellular space may be modelled as a distinct compartment with its own signal characteristics, but oriented identically to the intra-axonal compartment. Under this assumption, this shared orientation structure can be captured in a single orientation distribution P, and the microstructural characteristics can be modelled by a single voxel-level kernel H, whose signal response is the mixture of the constituent compartment responses.

Nearly all of the most common biophysical white matter models hence subsume an overarching *spherical convolution* model (Tournier et al., 2004; Anderson, 2005; Jespersen et al., 2007), in which the dMRI signal

(1) $$S\left(b,g\right)=\left(H*P\right)\left(b,g\right)=\int_{S^{2}}H\left(b,g\cdot n\right)\;P\left(n\right)\;\text{d}n.$$

The voxel-wise microstructure kernel H(b,θ) describes the signal response in straight, parallel white matter fascicles, as an axially-symmetric function of the *b*-value and of the cosine *θ* of the angle between the fibre orientation n and the diffusion gradient direction g. This voxel-level response can encapsulate the mixture of all compartments that share the same orientational structure, as well as any isotropic compartments. The (unnormalized) orientation distribution function P(n) captures the direction and dispersion of the different fascicles in the voxel, and is fundamental in tractography.

Different biophysical models adopt different constraints and functional forms for H and P, many of which have been heavily debated in the community (Jelescu and Budde, 2017; Novikov et al., 2018a), but can ultimately be written in the form of Eq. (1). The microstructure kernel is typically modelled as a mixture of restricted, hindered and fast water compartments, each parameterized by their axial and radial diffusivities and signal fractions (Behrens et al., 2003, 2007; Kroenke et al., 2004; Assaf and Basser, 2005; Jespersen et al., 2007, 2010; Alexander et al., 2010; Fieremans et al., 2011; Panagiotaki et al., 2012; Zhang et al., 2012; Ferizi et al., 2015; Jelescu et al., 2016; Kaden et al., 2016; Reisert et al., 2017; Novikov et al., 2018b). Other approaches have adopted empirical forms for the response kernel, in some cases invariant across the brain (Tournier et al., 2004; Anderson, 2005; Tournier et al., 2007; Dell’Acqua et al., 2007; Jian and Vemuri, 2007; Descoteaux et al., 2009; Jeurissen et al., 2014; Christiaens et al., 2017). The orientation structure is often represented as an orientation distribution function (ODF) in the spherical harmonics (SH) basis (Frank, 2002), as it is the most general representation and reduces the convolution in (1) to a multiplication of corresponding SH coefficients (Tournier et al., 2004, 2007; Jespersen et al., 2007, 2010; Reisert et al., 2017; Novikov et al., 2018b). Other models have selected functional descriptors of the orientation structure, for instance in the form of *δ*-functions or Watson-distributions representing one or several fibre populations and their dispersion (Behrens et al., 2003, 2007; Zhang et al., 2012; Sotiropoulos et al., 2012).

The large combinatorial space of different model choices for H and P, not to mention the many different fitting strategies, has led to a plethora of microstructure imaging techniques. The quest for the right model on the effective length scale in dMRI therefore critically depends on validating the underlying model assumptions *in vivo* (Novikov et al., 2018a). Most validation studies have focused on the functional form of the microstructure kernel H (Dhital et al., 2018; Veraart et al., 2019; Tax et al., 2019), but until now the assumed voxel-wise uniqueness of this kernel and its corresponding ODF has not been evaluated.

**Fig. 1:**
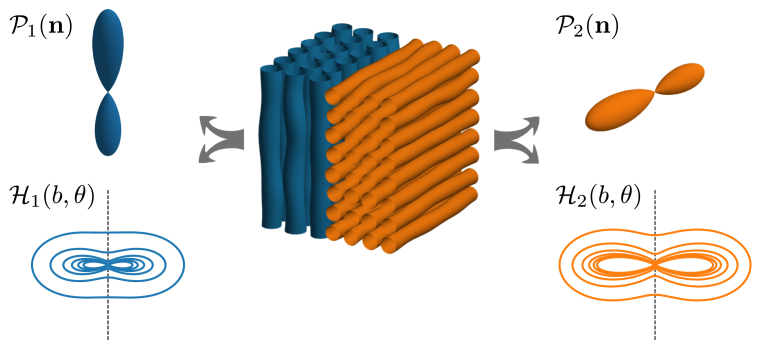
Sketch of a voxel where the microstructure is not described by a single rotation-invariant kernel, as most models assume. The blue, vertical white matter fascicles are modelled as the spherical convolution of microstructure kernel $H_{1}\left(b,\theta \right)$ and ODF $P_{1}\left(n\right)$. The orthogonal fascicles in orange are also modelled as a convolution (of $H_{2}\left(b,\theta \right)$ and $P_{2}\left(n\right)$), but with more restrictive diffusion resulting in a different microstructure kernel. This work validates if *in vivo* adult brain dMRI data can adequately be described as the convolution of a single microstructure kernel $H\left(b,\theta \right)\equiv H_{1}\left(b,\theta \right)\equiv H_{2}\left(b,\theta \right)$ and a single ODF $P\left(n\right)=P_{1}\left(n\right)+P_{2}\left(n\right)$.

Indeed, many of the most general models rely on the widely adopted assumption that, within an individual voxel, the microstructure-specific aspects can be expressed as a single kernel that can adequately represent all fascicles in that voxel. This implicit assumption of the convolution model in Eq. (1) imposes a clear simplification of the biology given that microstructural features might be fascicle-specific and that the coarse voxel size in dMRI inevitably introduces partial volume effects. Consider for example the fibre configuration sketched in Fig. 1, where two non-parallel fibre bundles have sufficiently different microstructural properties leading to them having different kernels $H_{1}$ and $H_{2}$. In this case, the total signal can generally not be described as a single convolution. To describe such a system requires a more generalized model with multiple convolutions, in this instance $S=H_{1}*P_{1}+H_{2}*P_{2}$, unless either both kernels or both ODFs are identical up to a normalization factor.

Note that, as per the equation above, multi-compartment models reduce to a single convolution if all directional compartments are coupled by a single ODF. Any isotropic compartments are trivially absorbed into a single kernel too. As such, the ball-and-stick model (Behrens et al., 2003, 2007), NODDI (Zhang et al., 2012), and other common 2- and 3-compartment models (Novikov et al., 2018b; Reisert et al., 2017), all abide by the single-convolution assumption. Multi-tissue constrained spherical deconvolution (MT-CSD) (Jeurissen et al., 2014) does too, as long as all but one response functions are isotropic. On the other hand, multi-tensor models (Tuch et al., 2002) and the CHARMED model (Assaf and Basser, 2005) where intra- and extra-axonal compartments are uncoupled in orientation are examples of models that do not assume the single-convolution form.

In this work, we will evaluate how well the *in vivo* dMRI signal in adult white matter is described by a single per-voxel response in even the most complex fibre configurations. Our approach is based on a voxel-wise decomposition into spherical harmonics and radial orthonormal components that, under the single-convolution model and in the absence of noise, offers a rank-1 descriptor of the dMRI signal (Christiaens et al., 2019). Testing the model rank *in vivo* can therefore validate the assumption of a single response per voxel, without imposing any prior constraints or functional form on the response or on the ODF. We will evaluate the relative effect size of higher-order signal components in rich multi-shell data of 4 healthy adult volunteers, and determine their significance with permutation testing. Source code and data are publicly available at https://osf.io/mjbnz.

## 2 Materials and methods

### 2.1 Data and preprocessing

Multi-shell dMRI data were acquired in 4 healthy adults (3 male, 1 female) on a 3T Philips Achieva system with 32-channel head coil. The multi-shell encoding scheme was designed to comprise 6 shells at b=900, 1600, 2500, 3600, 4900, and $6400\;\text{s}/{\text{mm}}^{2}$ (linear in $q\propto \sqrt{b}$) with 70 isotropically-distributed directions each and 30 b=0 volumes, all interleaved for minimal duty cycle (Hutter et al., 2017). Each volume is acquired with isotropic resolution 2.5 ​mm, matrix size 96×96×50 slices, TE=91ms, TR=6260ms, multiband factor 2, and SENSE factor 2. We also acquired a matched single-band b=0 reference scan with AP-PA and LR-RL phase encoding.

The dMRI data were first processed with patch-level image denoising based on random matrix theory in complex (magnitude and phase) data, which accounts for the noise correlations in most common dMRI examinations (Cordero-Grande et al., 2019). Since this step operates in the complex domain, effects of Rician bias are minimized in subsequent magnitude images. Mean SNR of the raw data (before denoising) was measured to be >30 at $b=0\;\text{s}/{\text{mm}}^{2}$ in white matter. The data was subsequently filtered for Gibbs ringing suppression (Kellner et al., 2016). The reverse phase encoding reference scan was used to estimate the B0 field map (Andersson et al., 2003), which was then used as part of subject motion and image distortion correction (Andersson and Sotiropoulos, 2016). Brain masks were segmented with FSL (Smith, 2002).

### 2.2 Voxel-wise decomposition

The spherical convolution (1) is conventionally projected onto the orthonormal basis of real, symmetric spherical harmonics $Y_{\ell }^{m}\left(n\right)$ of order $\ell =0,2,\ldots ,\ell_{\text{max}}$ and index m=−ℓ,…,ℓ. Here, we use a linear least-squares fit in the SH basis of order $\ell_{\text{max}}=8$ in all experiments. In this basis, and without loss of generality, convolution with a single response reduces to a multiplication

(2) $$s_{\ell ,b}^{m}=\sqrt{\frac{4\pi }{2\ell +1}}\;{\bar{h}}_{\ell ,b}\;p_{\ell }^{m}=h_{\ell ,b}\;p_{\ell }^{m},$$

where $s_{\ell ,b}^{m}$ are SH coefficients representing the dMRI signal in shell *b* and $p_{\ell }^{m}$ are SH coefficients representing the ODF. The axially-symmetric response kernel is represented by coefficients ${\bar{h}}_{\ell ,b}$ in the basis of *zonal* spherical harmonics $Y_{\ell }^{0}\left(n\right)$, or equivalently by scaled coefficients $h_{\ell ,b}$ that encompass the convolution normalization factor to simplify the notation.

With multi-shell data, we can arrange the voxel-wise signal coefficients of corresponding SH order ℓ in matrices

(3) $${\text{S}}_{\ell }=\left[\begin{array}{ccccc} s_{\ell ,b_{1}}^{-\ell } & \cdots & s_{\ell ,b_{1}}^{0} & \cdots & s_{\ell ,b_{1}}^{\ell }\\ \vdots & & \vdots & & \vdots \\ s_{\ell ,b_{k}}^{-\ell } & \cdots & s_{\ell ,b_{k}}^{0} & \cdots & s_{\ell ,b_{k}}^{\ell } \end{array}\right]\text{,}$$

where *k* shells are laid out in rows and their (2ℓ+1) SH coefficients are laid out in columns. The SH convolution (2) can then be written as the outer product ${\text{S}}_{\ell }=h_{\ell }\;p_{\ell }^{⊤}$ of vectors $h_{\ell }=\left[\;\cdots \;h_{\ell ,b}\;\cdots \right]$ (indexed in *b*) and $p_{\ell }=\left[\;\cdots \;p_{\ell }^{m}\;\cdots \right]$ (indexed in *m*). As such, with a single per-voxel response and in the absence of noise, we have

(4) $$\begin{array}{c} S=H*P\;↔\;\forall \ell :\;{\text{S}}_{\ell }=h_{\ell }\;p_{\ell }^{⊤} \end{array}$$

and by construction, the matrices ${\text{S}}_{\ell }$ have matrix rank =1 (Christiaens et al., 2019). If, on the other hand, the signal is not adequately described by a single rotation-invariant response, we can introduce a generalized model with multiple kernels and their corresponding ODFs:

(5) $$\begin{array}{c} S=\sum_{i=1}^{n}H_{i}*P_{i}\;↔\;\forall \ell :\;{\text{S}}_{\ell }=\sum_{i=1}^{n}h_{\ell ,i}p_{\ell ,i}^{⊤} \end{array}$$

and where ${\text{S}}_{\ell }$ are generally of rank >1. Therefore, the matrix rank of ${\text{S}}_{\ell }$ can reveal the presence of multiple microstructure kernels for different orientations in the voxel.

In the special case where all kernels $H_{i}$ are identical up to a scale factor ($H_{i}=\alpha_{i}\;H$), equation (5) reduces to the single-convolution model (with $P=\underset{i}{\sum }\alpha_{i}\;P_{i}$) due to distributivity of the convolution and matrix product operations. Similarly, when all ODFs are identical up to a scale factor ($P_{i}=\beta_{i}\;P$) the generalized model also reduces to the form of (4) (in this case with $H=\sum_{i}\beta_{i}\;H_{i}$), which can for instance arise when all fascicles are parallel. Furthermore, we also note that $\text{rank}\left({\text{S}}_{0}\right)\equiv 1$ in all voxels because matrices ${\text{S}}_{0}$ have only one column. This property reflects the observation that isotropic compartments can always be absorbed in a single per-voxel kernel: the ℓ=0 SH band offers no capability to disentangle multiple independent kernels and ODFs.

In this work, we use the singular value decomposition (SVD) of matrices ${\text{S}}_{\ell }$ as a means to determine the number of significant microstructure components. Specifically,

(6) $${\text{S}}_{\ell }=\overset{r}{\underset{i=1}{\sum \;}}u_{\ell ,i}\;\sigma_{\ell ,i}\;v_{\ell ,i}^{⊤}\text{,}$$

where $u_{\ell ,i}$ and $v_{\ell ,i}$ are the left and right singular vectors and $\sigma_{\ell ,i}$ are the singular values. This decomposition is a voxel-wise specialization of the spherical harmonics and radial decomposition (SHARD) representation introduced in Christiaens et al. (2019), and will generally be of full rank due to inevitable noise and biological variability in the data. By comparing this SVD to (4), it is clear that the leading left and right singular vectors $u_{\ell ,1}$ and $v_{\ell ,1}$ retrieve — up to a normalization factor — the predominant microstructure response and ODF respectively. The subsequent components i>1 span the orthogonal space, i.e., the axes of variability not captured in the leading component. The associated singular values offer a measure of the effect size of this unexplained variance, thus making it possible to determine which components contain meaningful structure.

### 2.3 Relative signal explained

The squared singular values $\sigma_{\ell ,i}^{2}$ measure the signal power of each component *i* in the SH band of order ℓ. In order to retrieve a single measure of each component’s total effect size, we compute the signal power across all harmonic bands

(7) $$\sigma_{i}^{2}=\underset{\ell =0,2,\ldots }{\sum }\sigma_{\ell ,i}^{2}\text{.}$$

Its square root $\sigma_{i}$ measures the root-mean-squared (RMS) signal in each component; it has the same units as the signal. The ratio

(8) $$R=\frac{\sigma_{1}^{2}}{\sum_{i}\sigma_{i}^{2}}\cdot 100\text{\%,}$$

then provides a measure of the signal power explained in the leading component. Under the rank-1 model, this ratio is expected to be near 100%. Mapping this measure across the brain thus enables to evaluate the proportion of the signal that is explained in the single-convolution model. Conversely, 100%−R measures the local residual signal not explained with a single spherical convolution.

### 2.4 Permutation testing

In order to evaluate if there is evidence for multiple (fascicle-specific) kernels in a voxel, we wish to assess the effect size of additional components in relation to a distribution of unstructured residuals. We formulate the null hypothesis as

H0 Components i>1 contain no meaningful structure.

We wish to evaluate the null hypothesis with as few prior assumptions as possible. To this end, we deploy a permutation testing strategy in which the null distribution is sampled numerically using residual bootstrapping. This process destroys any structure in the residuals of a rank-1 model by inducing random permutations and applying the appropriate leverage. Specifically, we first calculate the rank-1 representation $\hat{y}$ in each voxel and its residuals $e=y-\hat{y}$ to the dMRI signal y. Subsequently, we sample $N=10^{4}$ bootstrap instances, each of which is generated with the following procedure:

1. Apply a random permutation Π to the residuals and correct for leverage with a factor *h* (see below):

$$e’=\frac{1}{\sqrt{1-h}}\;\text{Π}\;e;$$

2. Construct bootstrap instance $y’=\hat{y}+e’$;
3. Fit bootstrap instance y’ into the SH basis, construct matrices ${\text{S}}_{\ell }$ and their singular values $\sigma {’}_{\ell ,i}$;
4. Store the component effect sizes $\sigma {’}_{i}$ of the bootstrap instance.

The histogram of $\sigma {’}_{i}$ determines the null distribution of the corresponding component *i*. The number of instances $N_{i}^{+}$ where the actual effect size in the data exceeds that of the randomized bootstrap, i.e., where $\sigma_{i}>\sigma {’}_{i}$, determines the voxel-wise *p*-values

(9) $$P_{i}=1-\frac{N_{i}^{+}+1}{N+1}\text{,}$$

of components i>1. $P_{2}$ then offers the primary metric of potential structure beyond the single-convolution model.

Correction for leverage is needed to rescale the variance of the residuals to the effective degrees of freedom (Cook and Weisberg, 1982). The mean squared residuals of a least-squares fit of a model with *κ* degrees of freedom to *ν* data points scales as $\frac{\nu -\kappa }{\nu }\;{\hat{s}}^{2}$, where ${\hat{s}}^{2}$ is the noise variance in the data. Hence, the root-mean-squared error of a least squares fit underestimates the actual noise level, and an inverse scaling is applied to compensate for this in permutation testing. For a well conditioned system, a leverage factor h=κ/ν achieves the appropriate scaling (Davidson, 2006). In our case, the total no. degrees of freedom in the model $\kappa =\sum_{\ell }k+\left(2\ell +1\right)-1=70$ (k=6 is the no. shells) and the no. data points ν=k×70=420, so h=1/6. We also verified in simulations that this definition approximates the effective noise scaling in a rank-1 SVD.

The per-voxel *p*-values $P_{i}$ are subsequently corrected for multiple comparisons using the Benjamini-Hochberg false discovery rate (FDR) procedure (Benjamini and Hochberg, 1995). FDR procedures control the proportion of false positives relative to the number of positive results, and are generally less stringent than family-wise error rate procedures such as Bonferroni correction. The specific FDR procedure used here (Benjamini-Hochberg, which assumes independent or positively correlated tests) is itself less stringent than other FDR approaches (Benjamini and Yekutieli, 2001). We adopt significance level α=0.05 and FDR=5%.

Note that the test statistic in components i>1 is affected by ℓ≥2 SH terms only, since the ℓ=0 SH term is always rank-1. Therefore, only anisotropic signal content (the contrast in the angular domain) contributes to the statistical evaluation.

## 3 Results

**Fig. 2:**
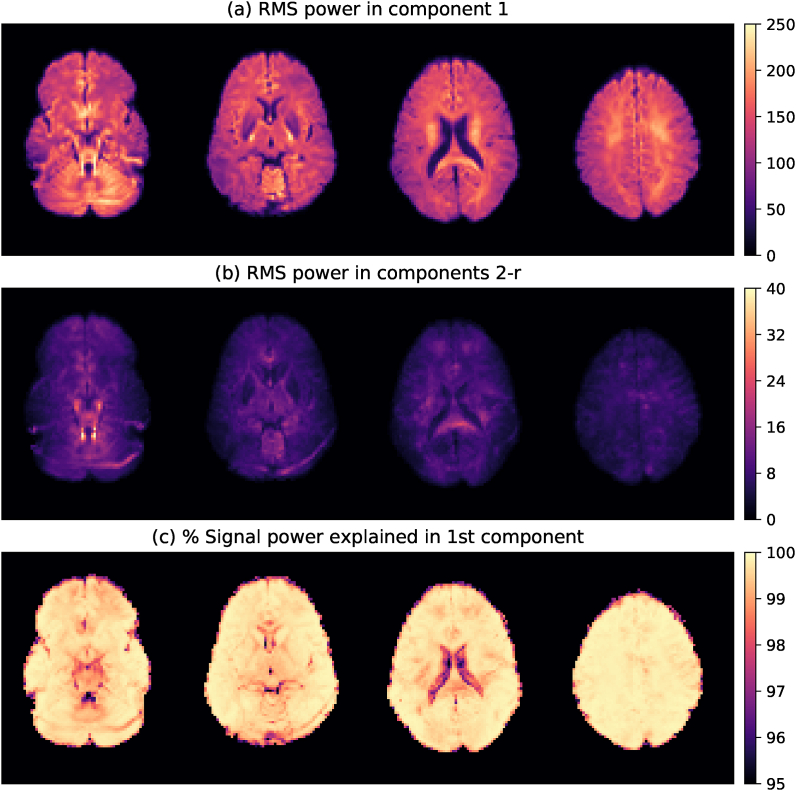
Signal power (ratio) in the leading component in Subject 4. (a) The root-mean-squared (RMS) signal power $\sigma_{1}$ in the principal voxel-wise SHARD component. (b) The total RMS signal power in components 2 to *r*, i.e., $\sqrt{{\sum^{\;}}_{i=2}^{r}\sigma_{i}^{2}}$. (c) The ratio *R* of the signal power explained in the first component. Supplementary Figures for the other subjects are provided online.

**Fig. 3:**
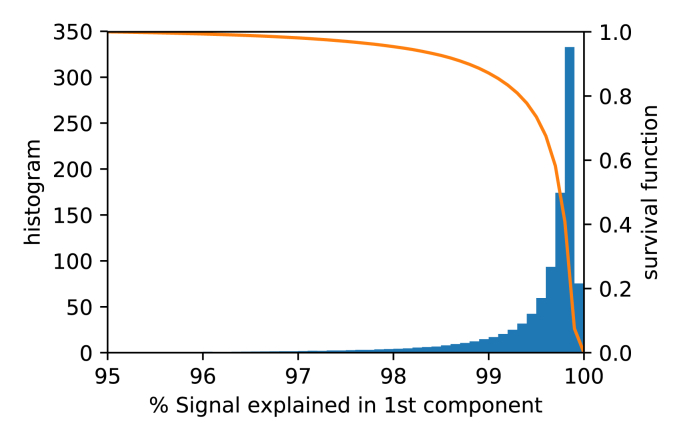
Histogram of the signal power explained in the first component across the full brain and across all 4 subjects (blue), and its survival function (1−CDF; orange curve). The plot shows that the data is well represented by the leading component alone.

Fig. 2a displays the RMS signal power $\sigma_{1}$ in the leading component. The total RMS power in the other components is shown in Fig. 2b. We observe that the leading component captures the main signal in the parenchyma. The other components, while not free of anatomical structure, primarily capture the overall noise modulation and image artefacts including a posterior fat shift artefact and flow or pulsation effects near the brain stem. Fig. 2c shows the ratio *R* of signal power explained in the leading component across the brain. We find that the rank-1 model explains >99% of the signal in the parenchyma, and observe no consistent reduction of this ratio in regions of crossing fibres. Similar figures for the other subjects are provided in supplementary materials and support this observation. A histogram of the % signal power explained in the rank-1 representation across all 4 subjects, shown in Fig. 3, confirms that the effect of additional components in a generalized model is small.

**Fig. 4:**
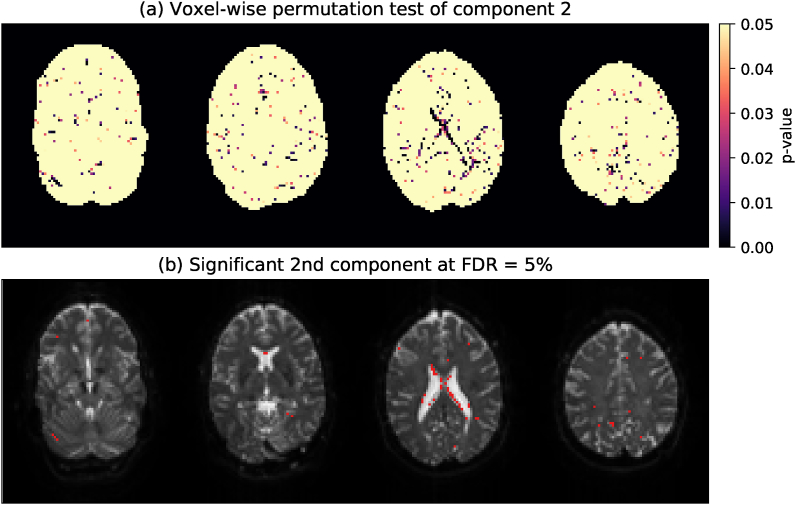
Permutation testing in Subject 2 to determine if component 2 contains any meaningful structure. (a) *p*-value of a voxel-wise residual bootstrapping test. (b) Significant voxels (α=0.05; shown in red) overlaid onto the mean b=0 image. Significance is determined after correcting for multiple comparisons using the Benjamini-Hochberg False Discovery Rate procedure with α=0.05. Supplementary Figures for the other subjects are provided online.

To determine if these additional components — however small — contain meaningful structure, we study the results of the permutation testing in Fig. 4. Fig. 4a shows the voxel-wise *p*-value of component 2 as estimated with the non-parametric residual bootstrapping procedure. We observe that $P_{2}<\alpha$ in scattered voxels with little discernible anatomical structure. After correcting for multiple comparisons in Fig. 4b, voxels with a significant 2nd component are increasingly sparse, with consistent anatomical structure only occurring at the interface between the ventricles and the corpus callosum where we do not expect multi-fascicle contributions. Visual inspection of the preprocessed dMRI data showed evidence of ventricular CSF pulsation, and previous studies have also pointed to effects of physiological noise in these regions (Walker et al., 2011). In components i≥3, no voxels survive multiple comparisons. Corresponding figures for the other subjects are provided as supplementary material. Generally, permutation testing did not find evidence for higher-order components, indicating that the single-convolution model is an appropriate description for these data.

## 4 Discussion

This paper has introduced a model-independent means of evaluating the single convolution assumption implicit to most biophysical models for white matter diffusion MRI, i.e., the assumption that the signal is sufficiently described with a single ODF and a single rotation-invariant response function per voxel. Our approach is independent of the functional form of the microstructure response and independent of the complexity of the fibre orientation distribution, relying instead on a matrix decomposition shown to be rank-1 under the single-convolution model. Results in adult brain data show that the effect size of any signal components beyond the single-convolution model is small (<1% of the signal) and that these additional components are not statistically significant when corrected for multiple comparisons. Therefore, even though different fascicles in a voxel may have different microstructural characteristics, we find no evidence of their observability in the diffusion MRI signal that would warrant the added complexity of multi-fascicle models in healthy adult white matter across the broad range of *b*-values sampled in these data.

In the given experimental setup, this conclusion stands for healthy *in vivo* adult brain diffusion MRI data acquired on a clinical system. Whether the single-convolution assumption is also sufficient to describe *ex vivo* and post mortem data, pediatric brain data, or pathology, remains an open question. Similarly, while our acquisition protocol was designed to collect rich multi-shell data well beyond clinically feasible scan time (47 ​min), higher SNR, even longer acquisitions or stronger MRI gradient systems might reveal evidence for additional components. In these cases, the presented method can be used to evaluate if the single-convolution assumption is appropriate for the subject group and data at hand. Given that the data in this study supersedes what is considered clinically practical, the evidence suggests that we can build on the single-convolution model to extract clinically applicable biomarkers of adult white matter microstructure.

Of note is that the voxel-wise decomposition is also directly applicable to generalized diffusion encoding waveforms, and to diffusion time and echo time dependent contrasts. Indeed, any orientation-dependent contrast can be projected in the SH basis and incorporated as additional rows in matrices (3), subject to the same rank-1 decomposition in the single-convolution model. Including generalized diffusion encoding or time dependence might hence add complementary contrast to detect and recover multiple kernels and ODFs, and may be the subject of future work. Here, we prioritised linear diffusion MRI across an extended *b*-value range as the most clinically relevant protocol to investigate at this point.

**Fig. 5:**
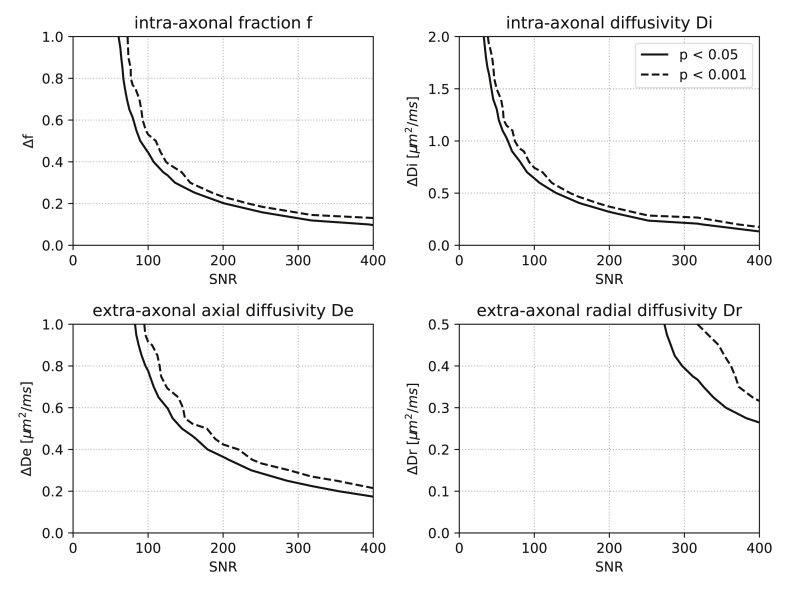
Simulations of the observability of kernel differences between two equally-weighted fascicles in a $90^{\circ }$ crossing. The curves plot the significance levels p<0.05 and p<0.001 of the bootstrapping test as a function of signal-to-noise ratio (SNR) and of differences in “standard model” parameters. For instance, when changing the intra-axonal fraction *f*, two kernels are simulated as $H_{stm}\left(\bar{f}\pm \frac{\text{Δ}f}{2},{\bar{D}}_{i},{\bar{D}}_{e},{\bar{D}}_{r}\right)$. The presence of multiple kernels is detected when their differences are large enough and when SNR if sufficiently high.

With the null result in mind, we now shine some light on how different the kernels need to be to break the single-convolution assumption at a given SNR. In Fig. 5, we show contour plots to demonstrate the SNR required to detect differences in standard model parameters between both fascicles (see also Appendix A: Simulations). With an asymptotic SNR ≈80 after denoising, the adult brain data in this work only allows the detection of strong differences between kernels, either in the intra-axonal fraction and diffusivity or in combinations of several parameters. This finding highlights that the single-convolution model is sufficient in most practical applications, even if the biological tissue properties are fascicle-specific.

Finally, while the presented model-free decomposition has a clear advantage encompassing a wide array of microstructure models, specific functional forms and constraints in model-based approaches may boost their statistical power. Multi-tensor models (Tuch et al., 2002; Scherrer and Warfield, 2010) are a case in point, where our generalized model is constrained to Gaussian responses and to ODFs in the form of SH *δ*-functions pointing along each fascicle direction. Whilst the multi-tensor model often reveals different diffusivities for the different fascicles in a voxel, these are driven in part by the fibre dispersion in each fascicle and in part by the non-mono-exponential signal response of the microstructure. Other examples include MT-CSD approaches with multiple anisotropic ODFs (Pietsch et al., 2019) that constrain the per-tissue responses to be invariant across the brain, and tractography-informed approaches (Reisert et al., 2014; Girard et al., 2017) that regularize microstructure parameters along fibre tracts. By using a local decomposition and deliberately avoiding functional forms, this work generalizes across a broad class of microstructure models. We can, however, not exclude that non-negativity or other sparsity-enforcing constraints on the ODF may reveal evidence for fascicle-specific microstructure kernels.

If, based on the evidence in this work, we accept the single-convolution assumption as valid, the proposed local SHARD decomposition has rank ​= ​1. This has interesting consequences for various applications. For instance, we could achieve voxel-level dMRI denoising, and potentially also outlier removal, by suppressing all but the leading component. Another interesting application can be in microstructure imaging based on rotation-invariant signal features (Reisert et al., 2017; Novikov et al., 2018b). Not only is the single-convolution assumption a prerequisite for using rotation-invariants in this way, the coefficients of the rank-1 decomposition can themselves form a set of rotation-invariant features useful in fitting a biophysical signal model.

## 5 Conclusion

While microstructural features are likely fascicle-specific, the diffusion MRI data in the white matter is well described by the spherical convolution of a single fibre orientation distribution and a single response function per voxel, regardless of their form and complexity. Our results suggest that we can build upon the current models of diffusion in adult white matter, despite the implicit single convolution assumption, and that a more complex model would not significantly improve the goodness-of-fit.

## Appendix A Simulations

In order to assess the sensitivity of our approach to kernel differences in a single voxel, we simulate a $90^{\circ }$ crossing of two fascicles as sketched in Fig. 1, each with their own microstructural properties. The ODFs $P_{1}$ and $P_{2}$ are defined as SH *δ*-functions of equal weight. The response functions $H_{1}$ and $H_{2}$ are simulated with a microstructural model, in this case the standard model of restricted intra-axonal and hindered extra-axonal diffusion (Novikov et al., 2018b):

(10) $$H_{stm}=f\;e^{-bD_{i}\theta^{2}}+\left(1-f\right)\;e^{-b\left(D_{e}\theta^{2}+D_{r}\left(1-\theta^{2}\right)\right)}\text{,}$$

where *f* is the intra-axonal volume fraction, $D_{i}$ is the intra-axonal (axial) diffusivity, $D_{e}$ is the extra-cellular axial diffusivity, $D_{r}$ is the extra-cellular radial diffusivity, and θ=g⋅n the cosine between the gradient direction and the fibre axis n. These simulations use *mean* parameter values $\bar{f}=0.5$, ${\bar{D}}_{i}=2.0\;\mu {\text{m}}^{2}/\text{ms}$, ${\bar{D}}_{e}=1.0\;\mu {\text{m}}^{2}/\text{ms}$ and ${\bar{D}}_{r}=0.5\;\mu {\text{m}}^{2}/\text{ms}$ and induce a symmetric change to each parameter. For example, changes in the intra-axonal fraction are induced by simulating $H_{1}$ with $\bar{f}+\frac{\text{Δ}f}{2}$ and $H_{2}$ with $\bar{f}-\frac{\text{Δ}f}{2}$. We vary Δf between 0 and 1, and the diffusivities $\text{Δ}D_{x}$ between 0 and ${\bar{D}}_{x}$.

The statistical assessment follows the permutation testing procedure described in Sec. 2.4, with the addition of Gaussian noise to each bootstrap instance (in Step 2). This evaluation is repeated for a dense grid of SNR and Δ values, resulting in a matrix of *p*-values for this grid. The contour lines at *p*-values 0.05 and 0.001, plotted in Fig. 5, then determine the minimum SNR and model parameter difference needed to detect a significant effect.

These results prove empirically that the proposed procedure detects the presence of multiple kernels when their differences are large enough and when SNR if sufficiently high. However, they also show that detecting realistic changes in SNR requires exceedingly high SNR. For example, the sensitivity is highest for changes in intra-axonal diffusivity $D_{i}$, but an SNR >100 is required to detect a relative difference as large as 30%. Changes in extra-axonal radial diffusivity $D_{r}$ are, for practical purposes, nearly impossible to detect. This confirms our conclusion that, while microstructural features may well be fascicle-specific, the diffusion MRI data in the white matter is well described by the single-convolution model. An important caveat is that, in contrast to the rest of the work, these simulations are by necessity model-based and may yield different results for more constrained signal representations.

## Appendix B Supplementary data

Multimedia component 1: Supplementary figures of the signal power (as shown in Fig. 2) and permutation testing results (as shown in Fig. 4) in the other subjects. Multimedia component 1

Research data for this article Research data for this article

## References

[bib1] Alexander D.C., Dyrby T.B., Nilsson M., Zhang H. (2019). Imaging brain microstructure with diffusion mri: practicality and applications. NMR Biomed..

[bib2] Alexander D.C., Hubbard P.L., Hall M.G., Moore E.A., Ptito M., Parker G.J., Dyrby T.B. (2010). Orientationally invariant indices of axon diameter and density from diffusion MRI. Neuroimage.

[bib3] Anderson A.W. (2005). Measurement of fiber orientation distributions using high angular resolution diffusion imaging. Magn. Reson. Med..

[bib4] Andersson J.L., Skare S., Ashburner J. (2003). How to correct susceptibility distortions in spin-echo echo-planar images: application to diffusion tensor imaging. Neuroimage.

[bib5] Andersson J.L., Sotiropoulos S.N. (2016). An integrated approach to correction for off-resonance effects and subject movement in diffusion MR imaging. Neuroimage.

[bib6] Assaf Y., Basser P.J. (2005). Composite hindered and restricted model of diffusion (CHARMED) MR imaging of the human brain. Neuroimage.

[bib7] Behrens T., Woolrich M., Jenkinson M., Johansen-Berg H., Nunes R., Clare S., Matthews P., Brady J., Smith S. (2003). Characterization and propagation of uncertainty in diffusion-weighted MR imaging. Magn. Reson. Med..

[bib8] Behrens T.E.J., Johansen-Berg H., Jbabdi S., Rushworth M.F.S., Woolrich M.W. (2007). Probabilistic diffusion tractography with multiple fibre orientations: what can we gain?. Neuroimage.

[bib9] Benjamini Y., Hochberg Y. (1995). Controlling the false discovery rate: a practical and powerful approach to multiple testing. J. R. Stat. Soc. Ser. B.

[bib10] Benjamini Y., Yekutieli D. (2001). The control of the false discovery rate in multiple testing under dependency. Ann. Stat..

[bib11] Callaghan P.T., Eccles C.D., Xia Y. (1988). NMR microscopy of dynamic displacements: k-space and q-space imaging. J. Phys. E Sci. Instrum..

[bib12] Christiaens D., Cordero-Grande L., Hutter J., Price A.N., Deprez M., Hajnal J.V., Tournier J.D. (2019). Learning compact q-space representations for multi-shell diffusion-weighted MRI. IEEE Trans. Med. Imaging.

[bib13] Christiaens D., Sunaert S., Suetens P., Maes F. (2017). Convexity-constrained and nonnegativity-constrained spherical factorization in diffusion-weighted imaging. Neuroimage.

[bib14] Cook R.D., Weisberg S. (1982). Residuals and Influence in Regression.

[bib15] Cordero-Grande L., Christiaens D., Hutter J., Price A.N., Hajnal J.V. (2019). Complex diffusion-weighted image estimation via matrix recovery under general noise models. Neuroimage.

[bib16] Davidson R., Patterson K., Mills T.C. (2006). Bootstrap methods in econometrics. Palgrave Handbooks of Econometrics.

[bib17] Dell’Acqua F., Rizzo G., Scifo P., Clarke R.A., Scotti G., Fazio F. (2007). A model-based deconvolution approach to solve fiber crossing in diffusion-weighted MR imaging. IEEE (Inst. Electr. Electron. Eng.) Trans. Biomed. Eng..

[bib18] Descoteaux M., Deriche R., Knosche T., Anwander A. (2009). Deterministic and probabilistic tractography based on complex fibre orientation distributions. IEEE Trans. Med. Imaging.

[bib19] Dhital B., Kellner E., Kiselev V.G., Reisert M. (2018). The absence of restricted water pool in brain white matter. Neuroimage.

[bib20] Ferizi U., Schneider T., Witzel T., Wald L.L., Zhang H., Wheeler-Kingshott C.A., Alexander D.C. (2015). White matter compartment models for in vivo diffusion MRI at 300 mT/m. Neuroimage.

[bib21] Fieremans E., Jensen J.H., Helpern J.A. (2011). White matter characterization with diffusional kurtosis imaging. Neuroimage.

[bib22] Frank L.R. (2002). Characterization of anisotropy in high angular resolution diffusion-weighted MRI. Magn. Reson. Med..

[bib23] Girard G., Daducci A., Petit L., Thiran J.P., Whittingstall K., Deriche R., Wassermann D., Descoteaux M. (2017). AxTract: toward microstructure informed tractography. Hum. Brain Mapp..

[bib24] Horsfield M.A., Jones D.K. (2002). Applications of diffusion-weighted and diffusion tensor mri to white matter diseases – a review. NMR Biomed.: An International Journal Devoted to the Development and Application of Magnetic Resonance In Vivo.

[bib25] Hutter J., Tournier J.D., Price A.N., Cordero-Grande L., Hughes E.J., Malik S., Steinweg J., Bastiani M., Sotiropoulos S.N., Jbabdi S., Andersson J., Edwards A.D., Hajnal J.V. (2017). Time-efficient and flexible design of optimized multishell HARDI diffusion. Magn. Reson. Med..

[bib26] Jelescu I.O., Budde M.D. (2017). Design and validation of diffusion MRI models of white matter. Frontiers in Physics.

[bib27] Jelescu I.O., Veraart J., Fieremans E., Novikov D.S. (2016). Degeneracy in model parameter estimation for multi-compartmental diffusion in neuronal tissue. NMR Biomed..

[bib28] Jespersen S.N., Bjarkam C.R., Nyengaard J.R., Chakravarty M.M., Hansen B., Vosegaard T., Østergaard L., Yablonskiy D., Nielsen N.C., Vestergaard-Poulsen P. (2010). Neurite density from magnetic resonance diffusion measurements at ultrahigh field: comparison with light microscopy and electron microscopy. Neuroimage.

[bib29] Jespersen S.N., Kroenke C.D., Østergaard L., Ackerman J.J., Yablonskiy D.A. (2007). Modeling dendrite density from magnetic resonance diffusion measurements. Neuroimage.

[bib30] Jeurissen B., Tournier J.D., Dhollander T., Connelly A., Sijbers J. (2014). Multi-tissue constrained spherical deconvolution for improved analysis of multi-shell diffusion MRI data. Neuroimage.

[bib31] Jian B., Vemuri B.C. (2007). A unified computational framework for deconvolution to reconstruct multiple fibers from diffusion weighted MRI. IEEE Trans. Med. Imaging.

[bib32] Kaden E., Kruggel F., Alexander D.C. (2016). Quantitative mapping of the per-axon diffusion coefficients in brain white matter. Magn. Reson. Med..

[bib33] Kellner E., Dhital B., Kiselev V.G., Reisert M. (2016). Gibbs-ringing artifact removal based on local subvoxel-shifts. Magn. Reson. Med..

[bib34] Kroenke C.D., Ackerman J.J., Yablonskiy D.A. (2004). On the nature of the NAA diffusion attenuated MR signal in the central nervous system. Magn. Reson. Med..

[bib35] Le Bihan D., Breton E., Lallemand D., Grenier P., Cabanis E., Laval-Jeantet M. (1986). MR imaging of intravoxel incoherent motions: application to diffusion and perfusion in neurologic disorders. Radiology.

[bib36] Neil J., Miller J., Mukherjee P., Hüppi P.S. (2002). Diffusion tensor imaging of normal and injured developing human brain – a technical review. NMR Biomed.: An International Journal Devoted to the Development and Application of Magnetic Resonance In Vivo.

[bib37] Novikov D.S., Fieremans E., Jespersen S.N., Kiselev V.G. (2019). Quantifying brain microstructure with diffusion MRI: theory and parameter estimation. NMR Biomed..

[bib38] Novikov D.S., Kiselev V.G., Jespersen S.N. (2018). On modeling. Magn. Reson. Med..

[bib39] Novikov D.S., Veraart J., Jelescu I.O., Fieremans E. (2018). Rotationally-invariant mapping of scalar and orientational metrics of neuronal microstructure with diffusion MRI. Neuroimage.

[bib40] Panagiotaki E., Schneider T., Siow B., Hall M.G., Lythgoe M.F., Alexander D.C. (2012). Compartment models of the diffusion MR signal in brain white matter: a taxonomy and comparison. Neuroimage.

[bib41] Pietsch M., Christiaens D., Hutter J., Cordero-Grande L., Price A.N., Hughes E., Edwards A.D., Hajnal J.V., Counsell S.J., Tournier J.D. (2019). A framework for multi-component analysis of diffusion MRI data over the neonatal period. Neuroimage.

[bib42] Reisert M., Kellner E., Dhital B., Hennig J., Kiselev V.G. (2017). Disentangling micro from mesostructure by diffusion MRI: a bayesian approach. Neuroimage.

[bib43] Reisert M., Kiselev V.G., Dihtal B., Kellner E., Novikov D.S., Golland P., Hata N., Barrilot C., Hornegger J., Howe R. (2014). MesoFT: unifying diffusion modelling and fiber tracking. Medical Image Computing and Computer-Assisted Intervention – MICCAI 2014.

[bib44] Scherrer B., Warfield S.K. (2010). Why multiple b-values are required for multi-tensor models. evaluation with a constrained log-euclidean model. 2010 IEEE International Symposium on Biomedical Imaging: from Nano to Macro.

[bib45] Smith S.M. (2002). Fast robust automated brain extraction. Hum. Brain Mapp..

[bib46] Sotiropoulos S.N., Behrens T.E., Jbabdi S. (2012). Ball and rackets: inferring fiber fanning from diffusion-weighted MRI. Neuroimage.

[bib47] Sullivan E.V., Pfefferbaum A. (2006). Diffusion tensor imaging and aging. Neurosci. Biobehav. Rev..

[bib48] Sundgren P., Dong Q., Gomez-Hassan D., Mukherji S., Maly P., Welsh R. (2004). Diffusion tensor imaging of the brain: review of clinical applications. Neuroradiology.

[bib49] Tax C.M., Szczepankiewicz F., Nilsson M., Jones D.K. (2019). The dot-compartment revealed? diffusion MRI with ultra-strong gradients and spherical tensor encoding in the living human brain. biorXiv.

[bib50] Tournier J.D., Calamante F., Connelly A. (2007). Robust determination of the fibre orientation distribution in diffusion MRI: non-negativity constrained super-resolved spherical deconvolution. Neuroimage.

[bib51] Tournier J.D., Calamante F., Gadian D.G., Connelly A. (2004). Direct estimation of the fiber orientation density function from diffusion-weighted MRI data using spherical deconvolution. Neuroimage.

[bib52] Tuch D.S., Reese T.G., Wiegell M.R., Makris N., Belliveau J.W., Wedeen V.J. (2002). High angular resolution diffusion imaging reveals intravoxel white matter fiber heterogeneity. Magn. Reson. Med..

[bib53] Veraart J., Fieremans E., Novikov D.S. (2019). On the scaling behavior of water diffusion in human brain white matter. Neuroimage.

[bib54] Walker L., Chang L.C., Koay C.G., Sharma N., Cohen L., Verma R., Pierpaoli C. (2011). Effects of physiological noise in population analysis of diffusion tensor MRI data. Neuroimage.

[bib55] Zhang H., Schneider T., Wheeler-Kingshott C.A., Alexander D.C. (2012). NODDI: practical in vivo neurite orientation dispersion and density imaging of the human brain. Neuroimage.

