# Supplementary material for: On the need for bundle-specific microstructure kernels in diffusion MRI
Source: Neuroimage. 2020 Mar;208:116460. doi: 10.1016/j.neuroimage.2019.116460 (PMC7014821; doi:10.1016/j.neuroimage.2019.116460)
Supplement: Multimedia component 1: — Supplementary figures of the signal power (as shown in Fig. 2) and permutation testing results (as shown in Fig. 4) in the other subjects. [file mmc1.pdf]

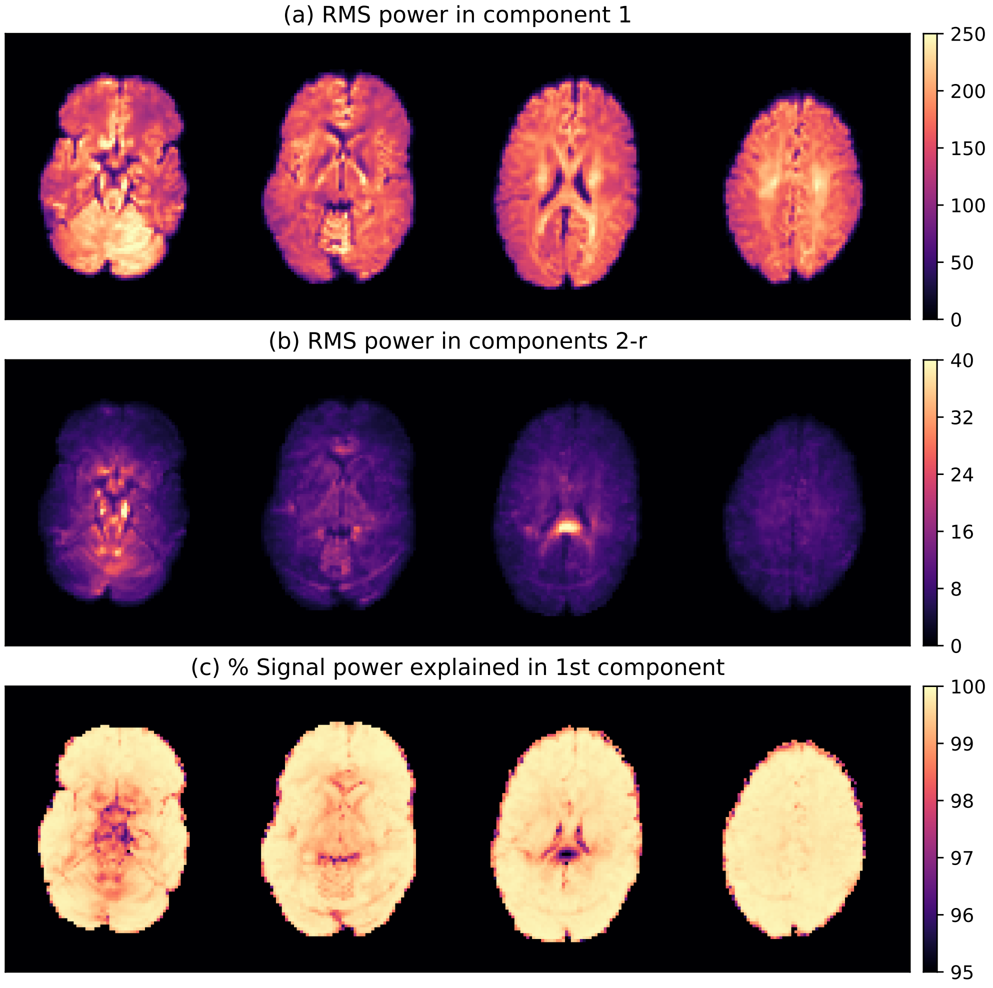

Figure S.1: Signal power (ratio) in the leading component in Subject 1. (a) The root-mean-squared (RMS) signal power σ_1 in the principal voxel-wise SHARD component. (b) The total RMS signal power in components 2 to r, i.e., $\sqrt{\sum_{i=2}^{r} \sigma_i^2}$. (c) The ratio R of the signal power explained in the first component.

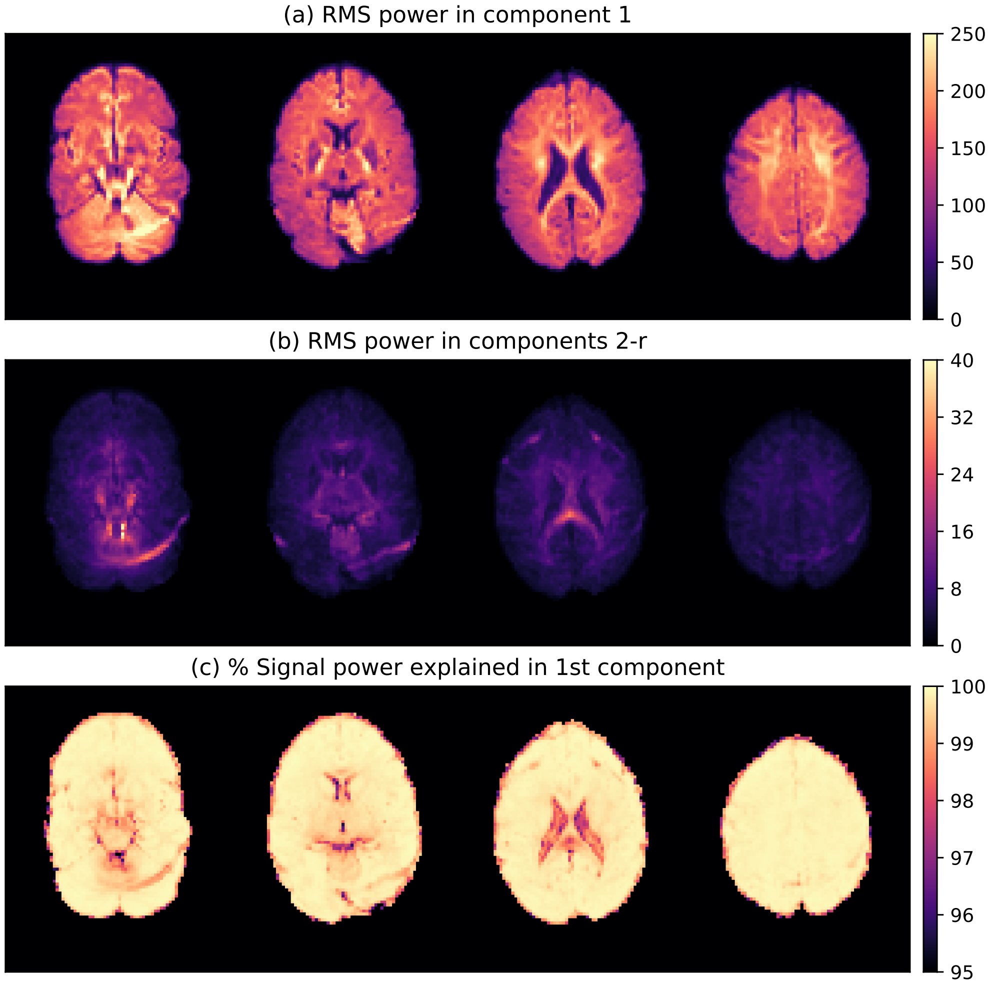

Figure S.2: Signal power (ratio) in the leading component in Subject 2. (a) The root-mean-squared (RMS) signal power σ_1 in the principal voxel-wise SHARD component. (b) The total RMS signal power in components 2 to r, i.e., $\sqrt{\sum_{i=2}^{r} \sigma_i^2}$. (c) The ratio R of the signal power explained in the first component.

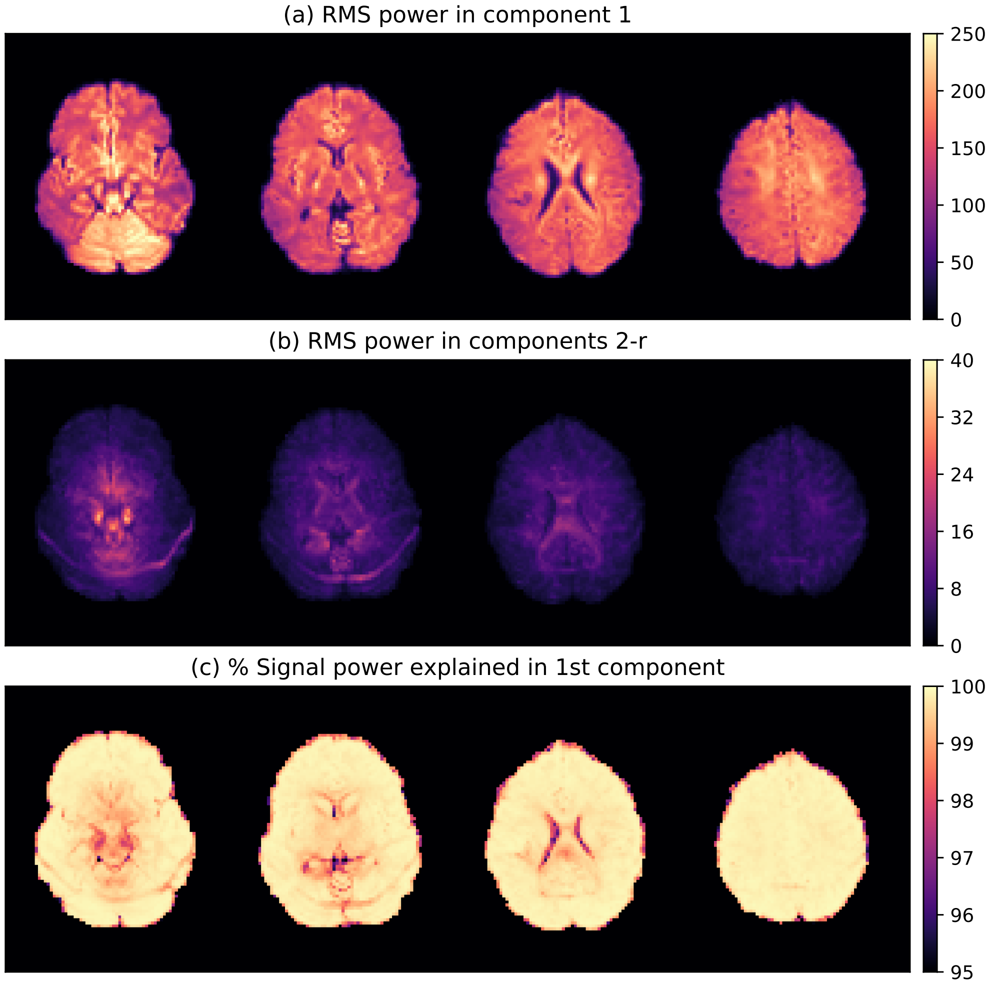

Figure S.3: Signal power (ratio) in the leading component in Subject 3. (a) The root-mean-squared (RMS) signal power σ_1 in the principal voxel-wise SHARD component. (b) The total RMS signal power in components 2 to r, i.e., $\sqrt{\sum_{i=2}^{r} \sigma_i^2}$. (c) The ratio R of the signal power explained in the first component.

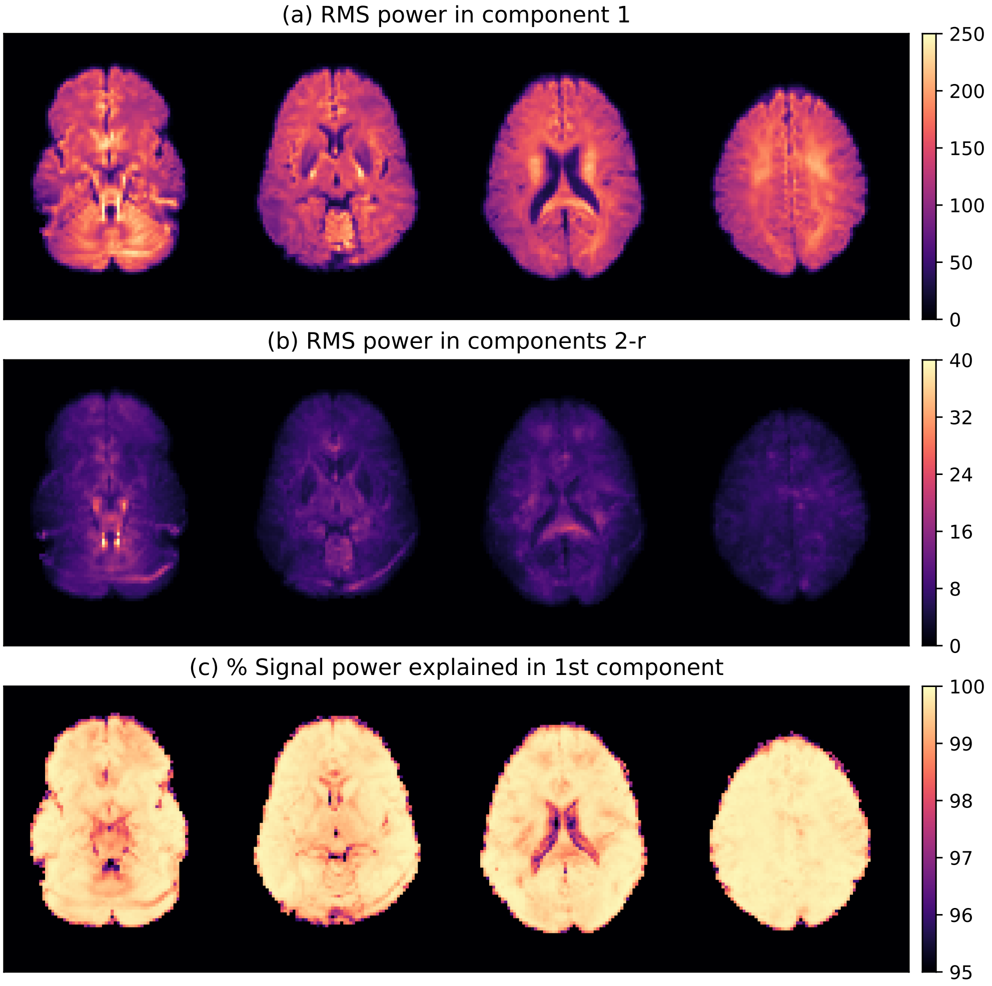

Figure S.4: Signal power (ratio) in the leading component in Subject 4. (a) The root-mean-squared (RMS) signal power σ_1 in the principal voxel-wise SHARD component. (b) The total RMS signal power in components 2 to r, i.e., $\sqrt{\sum_{i=2}^{r} \sigma_i^2}$. (c) The ratio R of the signal power explained in the first component.

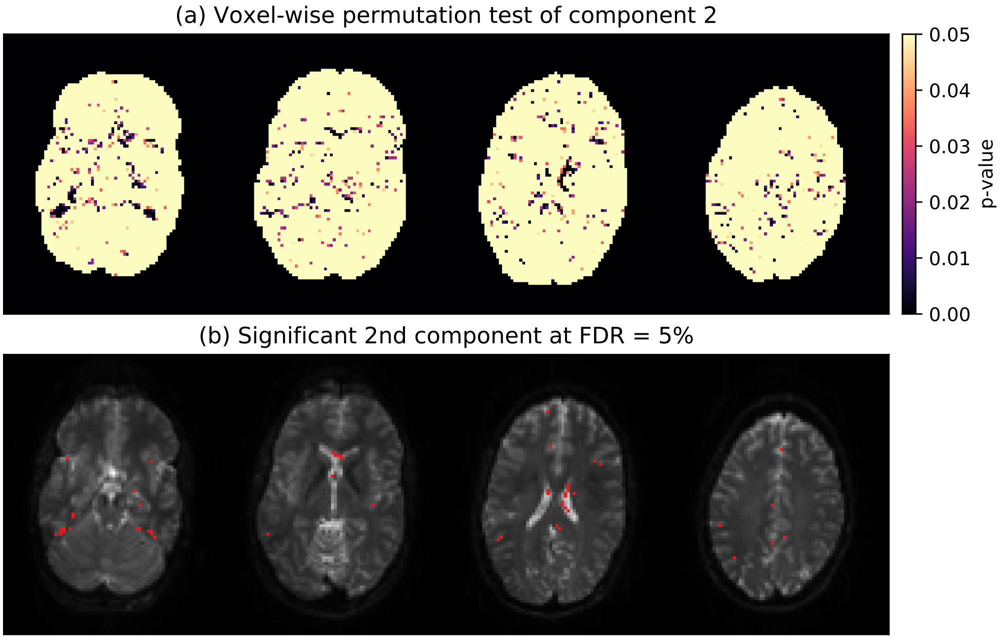

Figure S.5: Permutation testing in Subject 1 to determine if component 2 contains any meaningful structure. (a) p-value of a voxel-wise residual bootstrapping test. (b) Significant voxels ($\alpha = 0.05$; shown in red) overlaid onto the mean $b = 0$ image. Significance is determined after correcting for multiple comparisons using the Benjamini-Hochberg False Discovery Rate procedure with $\alpha = 0.05$.

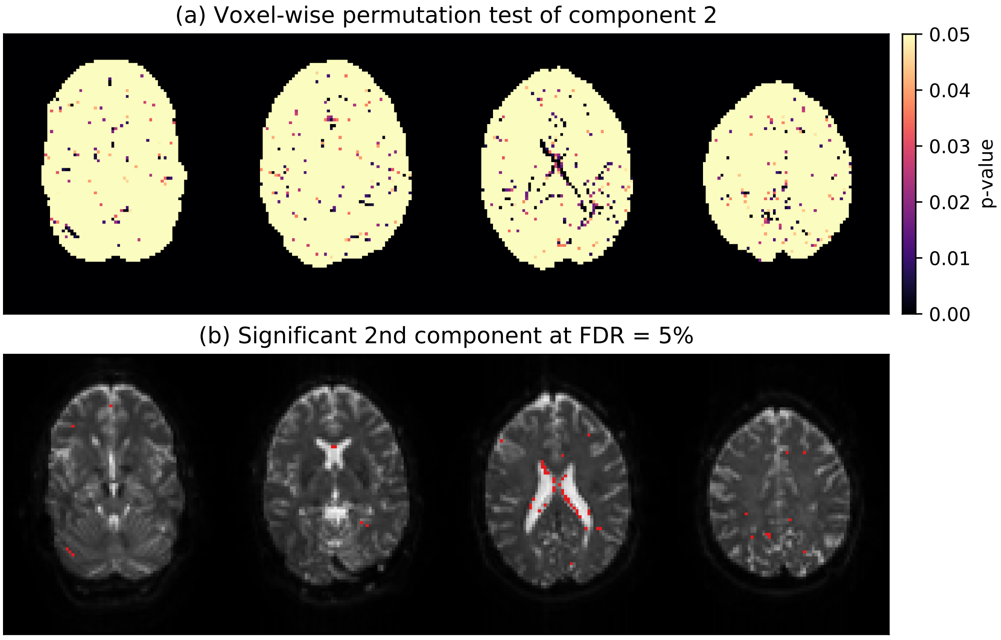

Figure S.6: Permutation testing in Subject 2 to determine if component 2 contains any meaningful structure. (a) p-value of a voxel-wise residual bootstrapping test. (b) Significant voxels ($\alpha = 0.05$; shown in red) overlaid onto the mean $b = 0$ image. Significance is determined after correcting for multiple comparisons using the Benjamini-Hochberg False Discovery Rate procedure with $\alpha = 0.05$.

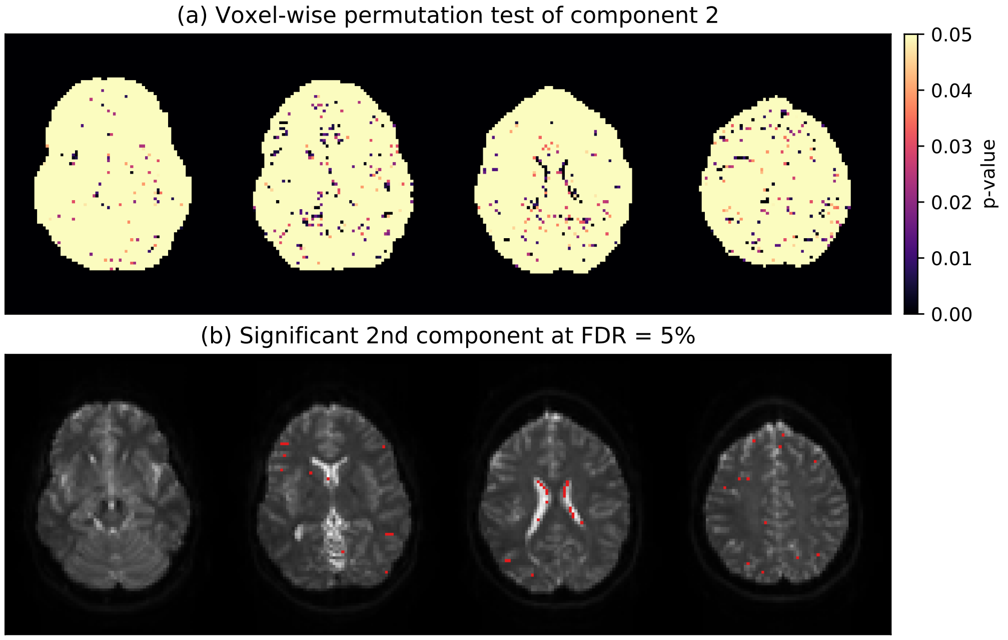

Figure S.7: Permutation testing in Subject 3 to determine if component 2 contains any meaningful structure. (a) p-value of a voxel-wise residual bootstrapping test. (b) Significant voxels ($\alpha = 0.05$; shown in red) overlaid onto the mean $b = 0$ image. Significance is determined after correcting for multiple comparisons using the Benjamini-Hochberg False Discovery Rate procedure with $\alpha = 0.05$.

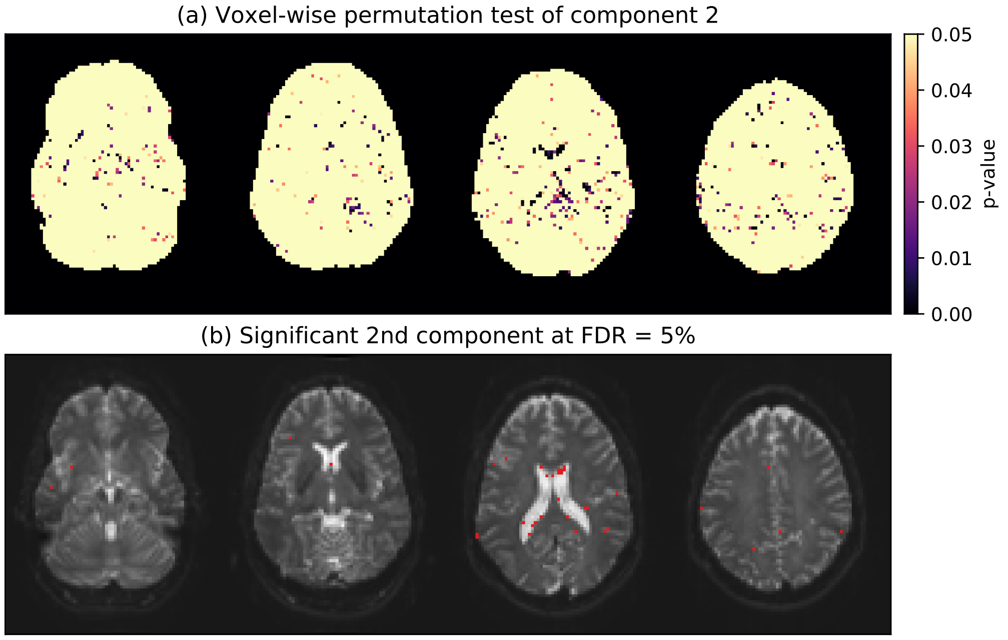

Figure S.8: Permutation testing in Subject 4 to determine if component 2 contains any meaningful structure. (a) p-value of a voxel-wise residual bootstrapping test. (b) Significant voxels ($\alpha = 0.05$; shown in red) overlaid onto the mean $b = 0$ image. Significance is determined after correcting for multiple comparisons using the Benjamini-Hochberg False Discovery Rate procedure with $\alpha = 0.05$.
